# Generation of RAG2 Knockout Immune-Deficient Miniature Pigs

**DOI:** 10.3390/ani14172597

**Published:** 2024-09-06

**Authors:** Jing Wang, Feiyan Zhu, Deling Jiao, Chang Yang, Junqi Wang, Fengchong Wang, Heng Zhao, Hong-Jiang Wei, Hong-Ye Zhao

**Affiliations:** 1 Yunnan Province Key Laboratory for Porcine Gene Editing and Xenotransplantation, Yunnan Agricultural University, Kunming 650201, China; kmwangjing@163.com (J.W.); yunnanfeiyan@163.com (F.Z.); jiaodeling@163.com (D.J.); theurgytheurgy@163.com (C.Y.); wangjunqi202209@163.com (J.W.); wfc8758369@163.com (F.W.); hengzhao2014@126.com (H.Z.); 2 Yunnan Province Xenotransplantation Research Engineering Centre, Yunnan Agricultural University, Kunming 650201, China; 3 Faculty of Animal Science and Technology, Yunnan Agricultural University, Kunming 650201, China; 4 College of Veterinary Medicine, Yunnan Agricultural University, Kunming 650201, China

**Keywords:** RAG2, knockout, severe immunodeficiency, pigs

## Abstract

**Simple Summary:**

Recombination-activating genes (RAGs) play a crucial role in the V(D)J recombination process. In this study, we generated RAG2 knockout (KO) pigs and evaluated the impact of RAG2 KO on the development of immune organs and immune cells. The thymus and spleen sizes of RAG2 KO pigs were significantly smaller than those of wild-type (WT) pigs, and their structures were highly disorganized and devoid of notable characteristic structures, indicating that RAG2 KO leads to dysplasia of the organs. The proportion of mature T and B lymphocytes in the circulation of RAG2 KO pigs was significantly reduced as compared to WT pigs. These findings successfully verified the immunodeficiency phenotype of RAG2 KO pigs. This study may provide immunodeficient animals for the development of tumor models and humanized animals.

**Abstract:**

Recombination-activating genes (RAGs) play a crucial role in the V(D)J recombination process and the development of immune cells. The development of the immune system and its mechanisms in pigs exhibit greater similarity to those of humans compared to other animals, thus rendering pigs a valuable tool for biomedical research. In this study, we utilized CRISPR/Cas9 gene editing and somatic cell nuclear transfer technology to generate RAG2 knockout (KO) pigs. Furthermore, we evaluated the impact of RAG2 KO on the immune organs and immune cell development through morphological observations, blood analysis and flow cytometry technology. RAG2 KO cell lines were used as donors for cloning. The reconstructed embryos were transplanted into 4 surrogate sows, and after 116 days of gestation, 2 sows gave birth to 12 live piglets, all of which were confirmed to be RAG2 KO. The thymus and spleen sizes of RAG2 KO pigs were significantly smaller than those of wild-type (WT) pigs. Hematoxylin-eosin staining results revealed that the thymus and spleen tissue structures of RAG2 KO pigs were disorganized and lacked the characteristic structures, indicating that RAG2 KO leads to dysplasia of the thymus and spleen. Hematological analysis demonstrated that the total number of white blood cells and lymphocytes in the circulation of RAG2 KO pigs was significantly lower, while the number of eosinophils was higher. Flow cytometry results indicated that the proportions of mature T and B lymphocytes were significantly reduced compared to WT pigs. These findings successfully verified the immunodeficiency phenotype of RAG2 KO pigs. This study may provide experimental animals for the development of tumor models and humanized animals.

## 1. Introduction

Severe combined immunodeficiency (SCID) is a genetic disorder characterized by impaired differentiation of T lymphocytes, developmental abnormalities of B cells and natural killer cells (NKs), and predominantly reduced or absent immune function. The initial clinical symptoms of SCID typically occur in the first few months of life, with the average age at diagnosis being 4–7 months. Epidemiological studies have demonstrated that the overall incidence of neonatal SCID is approximately 1/40,000 to 1/75,000 [1,2], and all types of SCID are fatal unless properly treated.

The mechanisms associated with immunodeficiency primarily encompass cytokine signaling pathways, V(D)J rearrangement, damage to T cell precursor cell receptors and increased lymphocyte apoptosis. Various immune-deficient mice targeting the key genes of the aforementioned mechanisms, such as SCID, NOD-SCID, NSG and NOG, have been constructed [3]. RAG1/2 play an important role in the V(D)J recombination process. RAG1 and RAG2 proteins are involved in the development of pre-mature T/B cells into mature T/B cells and initiate V(D)J recombination by recognizing the Ig or TCR gene and combining with the recombination signal sequence (RSS) in the gene fragment [4]. RAG1 and RAG2 genes are indispensable in the process of lymphocyte V(D)J rearrangement. The deletion of either gene results in the interruption of T and B lymphocyte development, which is characterized by severe early developmental block of T/B cells, leading to the inability to produce mature T/B lymphocytes [5,6] and the manifestation of symptoms similar to those of SCID in humans.

Homozygous deletion of RAG1/2 gene in mice has significantly reduced the T cells and B cells in the peripheral blood [7,8]. These mice are suitable for homogeneous and xenogeneic tumor transplantation, particularly when slow-growing, primary cells and blood-borne cancer cells need to be transplanted. They are more appropriate than nude mice, have better radiation tolerance than mice with SCID mutations and serve as an effective tool for tumor research. Immune-deficient mice are essential tools in immunodeficiency diseases, oncology research, and the construction and study of humanized mice. Nevertheless, the limitations of using mice as experimental animals have become increasingly apparent. The limitations of mice in tumor research are attributed to their small size and fragile tissues and organs, which make them unsuitable for surgical procedures and minimally invasive interventional treatments of cancer. Furthermore, the metabolism of mice is quite different from that of humans, making it challenging to simulate the heterogeneity of human tumors [9,10]. Many anti-cancer drugs that demonstrate efficacy in mouse tumor models fail in clinical trials, resulting in a low clinical translation rate of anti-cancer drugs [11,12,13].

Although large animals are more costly than rodents, studies on large animals can yield higher-quality research data and better prediction of the performance of new treatments and drugs in humans, and large animals should be considered as alternative animal models in tracking/follow-up studies [14]. Pigs are more similar to humans in body size, anatomy, genetics and immunology [15] and can provide blood samples at different stages of tumor onset, making it possible to study the entire course of tumors [16]. Consequently, immunodeficient pigs serve as superior animal models for preclinical safety and efficacy assessment of tumor treatment strategies [17,18,19]. In the study of immune-deficient pigs, various SCID pigs with deletion of IL2RG [20], RAG2 [21], RAG2/IL2RG [22] and ART^−/−^ IL2RG^−/Y^ [23] have been reported. However, the common issue is that these pigs have a short survival time. Regarding the application of SCID pigs, studies have reported the successful construction of melanoma [24], ovarian cancer [25] and osteosarcoma [26] in immunodeficient pigs. However, the application of immunodeficient pigs in the construction of human tumor models is limited by their short survival time, and further research is still needed on the construction of SCID pigs.

In addition, constructing immune system-humanized pigs is also an important application of SCID pigs for the study of human immune system diseases. The SCID mouse is widely used because of the generation of the humanized model in which HSCs cells are induced in a given SCID host to permit the differentiation of elements involved in the immune system [27,28]. However, the humanized SCID mouse also has certain limitations. For instance, mouse phagocytes directly involved in the killing of evolving human NK cell ancestors or non-recognition by mouse cytokines are needed for lineage maturation of NK cells. Due to the aforementioned limitations, researchers incline towards using large animals. As previously discussed, numerous studies have demonstrated that pigs are the most efficient models for biomedical research due to their considerable resemblance to humans. Research has reported that human hematopoietic stem cells injected into the intraperitoneal space of ART^−/−^ IL2RG^−/Y^ SCID pig fetuses were used to construct humanized pig models [23].

In this study, CRISPR/Cas9 gene editing technology in combination with somatic cell nuclear transfer technology was employed to generate RAG2 KO *Diannan* miniature pigs. Hematological and histopathological analyses were performed to evaluate the development of immune cells and immune organs in these gene-edited pigs. The immune-deficiency phenotype was verified by measuring the ratio of T and B lymphocytes in peripheral blood PBMCs by flow cytometry. This study may provide a large experimental animal model for investigating immunodeficiency diseases and facilitating the development of tumor models and humanized animals.

## 2. Materials and Methods

### 2.1. Laboratory Animals and Cell Lines

The RAG2 KO *Diannan* miniature pig fibroblast cell lines [29] and surrogate sows used in this study were obtained from the Key Laboratory for Porcine Gene Editing and Xenotransplantation in Yunnan Province, Yunnan Agricultural University. Primer synthesis and Sanger sequencing were completed by Beijing Tingke Biotech Co., Ltd., Beijing, China.

### 2.2. Somatic Cell Nuclear Transfer and Embryo Transfer 

Oocyte collection, in vitro maturation, SCNT and embryo transfer were performed as described previously [30]. Briefly, cultured cumulus-oocyte complexes (COCs) were isolated from cumulus cells by treating them with 0.1% (*w*/*v*) hyaluronidase. The first polar body was enucleated via gentle aspiration using a beveled pipette in TLH-PVA, while the donor cells were injected into the perivitelline space of the enucleated oocytes. The reconstructed embryos were fused with a single direct current pulse of 200 V/mm for 20 µs using the Electro Cell Fusion Generator (LF201, NEPA GENE Co., Ltd., Tokyo, Japan) in fusion medium. Embryos were then cultured in PZM-3 for 0.5–1 h and activated with a single pulse of 150 V/mm for 100 ms in activation medium. The embryos were equilibrated in PZM-3 supplemented with 5 µg/mL cytochalasin B for 2 h at 38.5 °C in a humidified atmosphere with 5% CO_2_, 5% O_2_ and 90% N_2_ (APM-30D, ASTEC, Fukuoka, Japan) and then cultured in PZM-3 medium under the same culture conditions described above until embryo transfer. The SCNT embryos were surgically transferred into the oviducts of the recipients, and piglets were obtained through natural delivery. 

### 2.3. Genotyping of RAG2 KO Piglets

The DNA was extracted from the muscle tissue cloned piglets, and the extracted tissue were used as templates to perform PCR amplification of the RAG2 gene using primers, as in our previous study [29]. RAG2 KO pigs were further genotyped by Sanger sequencing.

### 2.4. Routine Blood Tests

Blood samples from RAG2 KO and WT piglets were collected and used for routine blood testing comprising leukocytes, lymphocytes, basophils, neutrophils, monocytes and eosinophils. Blood routine was detected by an automatic hematology analyzer (BC-5000Vet, Mindray, Shenzhen, China).

### 2.5. Morphological Analysis of Immune Organs

Tissue samples of the thymus and spleen were collected surgically from the RAG2 KO pig and WT pigs and grossly analyzed. For HE staining, tissue samples were fixed in 4% paraformaldehyde for 48–72 h, processed by an automatic tissue processor (Yd-12p, Jinhua Yidi, medical appliance Co., Ltd., Jinhua City, China) and embedded in a paraffin block (Yd-6D, Jinhua Yidi, medical appliance Co., Ltd., Jinhua City, China). The paraffin blocks were cut into sections (5 μm) using a Microm HM 325 microtome (Thermo Scientific, Waltham, MA, USA) and allowed to dry on glass slides overnight at 37 °C. Afterwards, the tissue sections were deparaffinized in xylene and rehydrated through graded ethanol dilutions. The sections were stained with hematoxylin-eosin (H&E) (cat. no. G1120, Solarbio, Beijing, China) according to the manufacturer’s instructions. The slides were observed under the microscope (BX53, Olympus, Tokyo, Japan).

### 2.6. Immune Cell Flow Cytometry

All flow cytometry assays were performed by a CytoFlex flow cytometer (Beckman Coulter, Brea, CA, USA). For detection of T cells and B cells in PBMCs derived from RAG2 KO and WT pigs, 1 × 10^5^ cells were incubated with fluorescently labeled flow cytometry antibodies—CD3-FITC (cat. no. 559582, BD, Franklin Lakes, NJ, USA), CD45RA-APC (cat. no. 983004, Bio Legend, San Diego, CA, USA) and MONOCYIE/GRANULOCYTE panel-PE (cat. no. MA5-28824, ThermoFisher, Waltham, MA, USA)—for 15 min at 37 °C in the dark. After that, the cells were washed twice with PBS and centrifuged at 1400 rpm for 5 min. All data acquired from the flow cytometry were analyzed using Flow Jo 10.8 software.

### 2.7. Statistical Analysis

Statistical analysis was performed using SPSS 20.0 (IBM, New York, NY, USA). Independent-sample *t*-test were used to analyze the data. Data were expressed as means ± standard deviations of the means (SDs). Statistical significance was set at * *p* < 0.05 or ** *p* < 0.01.

## 3. Results

### 3.1. Generation of RAG2 KO Pigs

In our previous study [29], we obtained RAG2 KO fibroblast cells. The RAG2 gene was knocked out by the CRISPR/Cas9 system, a 1 bp insertion mutation and a 4 bp deletion mutation were detected in RAG2 KO fibroblast cells. And the already generated RAG2 KO fibroblast cells were used as donor cells for somatic cell cloning to obtain reconstructed embryos. The embryos were then surgically transplanted into recipients (*n* = 4), which received a total of 2680 embryos. The pregnancy status of the surrogate sows was detected by B-ultrasound on 23 days after embryo transplantation; 2 surrogate sows were pregnant (pregnancy rate = 50%); and 12 live piglets were obtained after natural delivery (Table 1).

### 3.2. Genotyping of RAG2 KO Piglets

**Figure 1:**
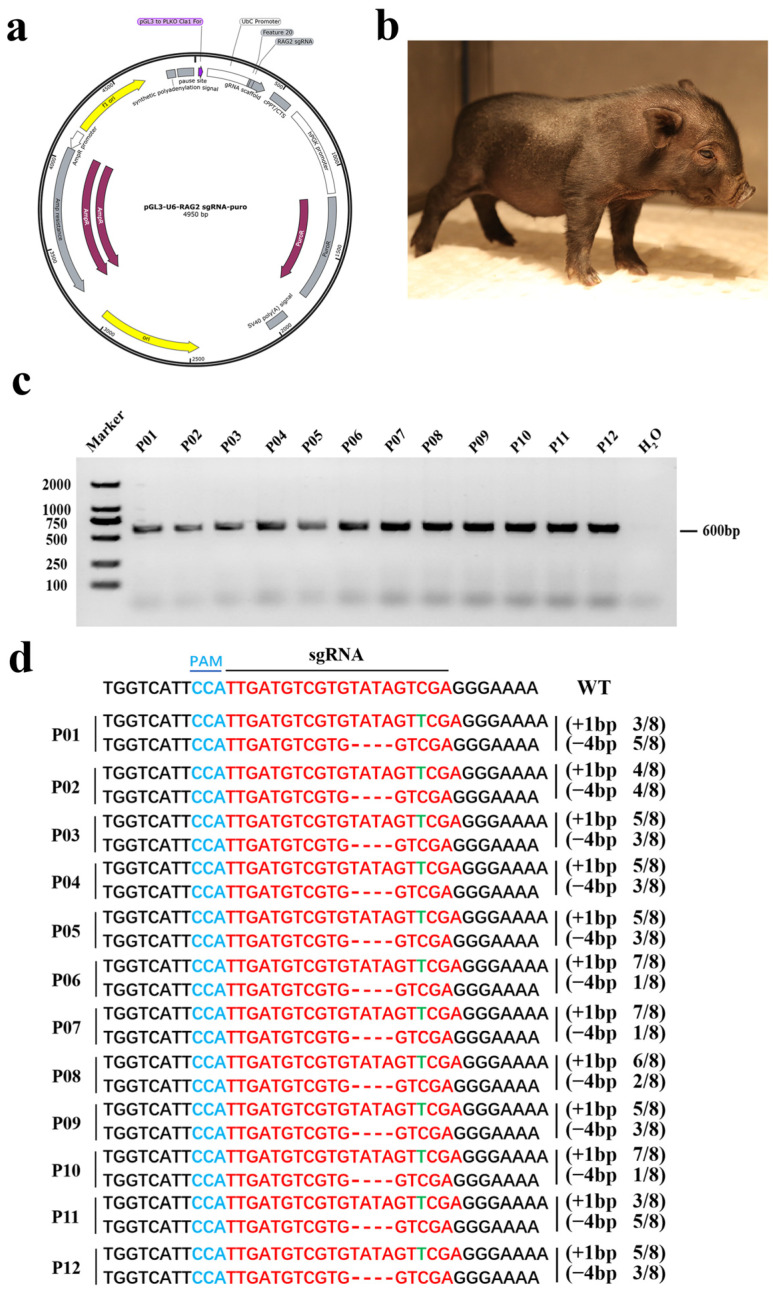
The generation and genotyping of RAG2 KO piglets. (**a**) The RAG 2 gene targeting vector. (**b**) Picture of an 8-day-old RAG2 KO piglet. (**c**) RAG2 KO piglets were genotyped by PCR (M: DL 2000 DNA marker; H_2_O: water). (**d**) Sanger sequencing results for RAG2 KO piglets. sgRNA was highlighted in red. The PAM structure was highlighted in blue. The insertion base was highlighted in green. (“−”: deletion, “+”: insertion, N/N: proportion of positive clones, WT: wild type).

We next identified the genotype of the targeted gene in the cloned piglets (Figure 1b), and the results showed that all the piglets carried a positive band of 600 bp (Figure 1c). The PCR products were sequenced through Sanger sequencing, and the results demonstrated that the genotypes of the 12 gene-edited piglets were consistent. The genomic sequencing showed that a 1 bp insertion mutation and a 4 bp deletion mutation were detected in RAG2 (RAG2 +1/−4) piglets (Figure 1d). Both mutations were sufficient to cause frame-shift mutations in the RAG2 gene, resulting in inactivtion RAG2 gene. 

### 3.3. RAG2 KO Affected the Development of the Thymus and the Spleen

**Figure 2:**
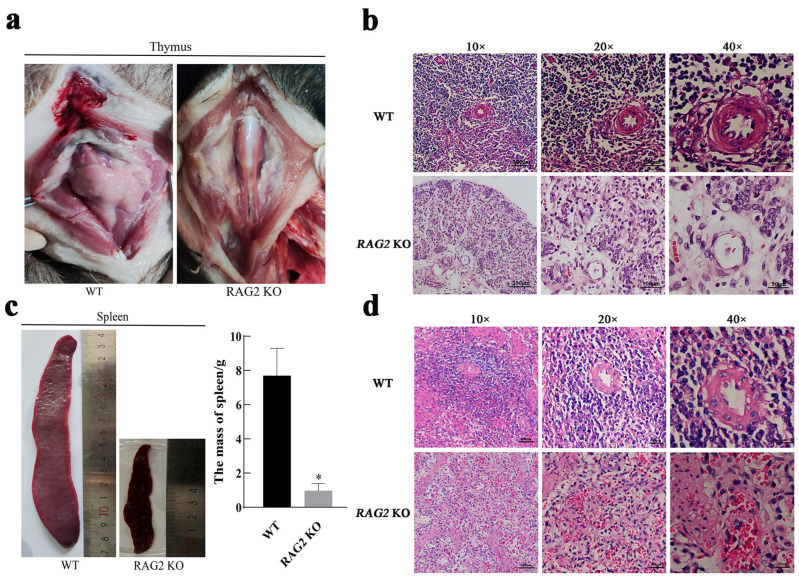
Effects of RAG2 gene knockout on thymus and spleen development. (**a**,**b**) The thymus gross and histomorphology of 1-month-old wild-type and RAG2 KO pigs. (**c**,**d**) The spleen morphology, mass and histomorphology of 1-month-old wild-type and RAG2 KO pigs. * *p* < 0.05 indicates a significant difference.

To compare the gross anatomy/morphology, the immune organs of RAG2 KO pigs and WT pigs of the same age were selected, revealing that knockout of the RAG2 gene resulted in hypoplasia of the thymus (Figure 2a) and significantly reduced spleen size and mass (Figure 2c). The thymus is composed of an outer cortex, primarily comprising epithelial cells, degenerated keratinized epithelial cells, myoid tissue, thymic lymphocytes and B lymphocytes. Among these components, B lymphocytes can form germinal centers. H&E staining of thymus tissue showed that obvious germinal centers and thymic corpuscles were visible in the thymus tissue of WT pigs, while the thymus tissue of RAG2 KO pigs was disorganized, with no obvious peculiar structures, corpuscles, or germinal centers. These observations suggested that RAG2 knockout led to thymic dysplasia. The spleen is the largest lymphoid organ in the human body. The parenchyma of the spleen is divided into three parts: white pulp, red pulp and a marginal zone. Similarly, the texture of the spleen tissue in RAG2 KO pigs was disorganized as compared with WT tissue. The central arterial duct in the red pulp of the spleen was absent and the edge zone transition was blurred. The results indicated that RAG2 knockout also affected the development of the spleen (Figure 2d).

### 3.4. Effects of RAG2 Gene Knockout on the Development of Immune Cells

**Figure 3:**
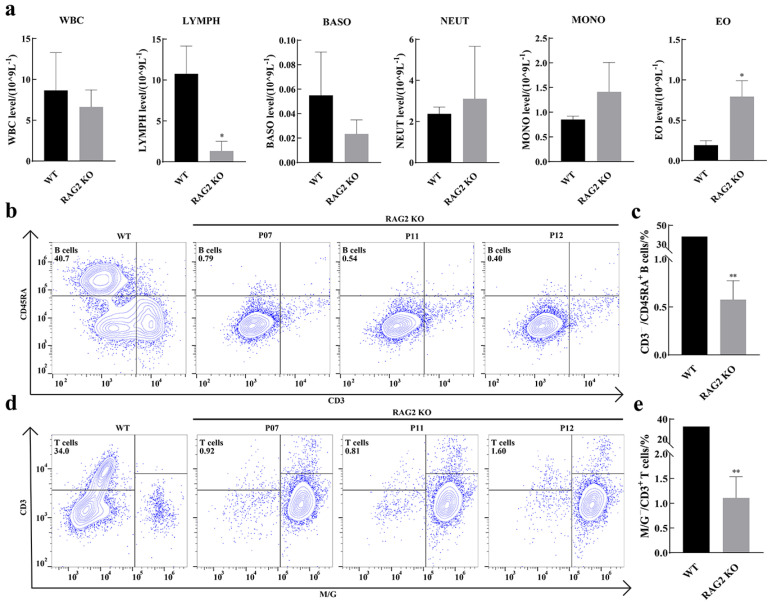
Effects of RAG2 knockout on the development of immune cells. (**a**) White blood cell (WBC), lymphocyte (LYMPH), basophil granulocyte (BASO), central granulocyte cell (NEUT), monocyte cell (MONO) and eosinophil granulocyte cell (EO) counts of WT and RAG2 KO piglets in the peripheral blood. (**b**,**c**) Percentage of mature B cells in PBMCs of WT and RAG2 KO piglets. (**d**,**e**) Percentage of mature T cells in PBMCs of WT and RAG2 KO piglets. (WT: wild type; * *p* < 0.05 or ** *p* < 0.01 indicates a significant difference (*n* = 3).).

To determine the effect of RAG2 KO on immune cells from the peripheral blood of pigs, we routinely tested the numbers of leukocytes, lymphocytes, basophils, neutrophils, monocytes and eosinophils in the peripheral blood (Figure 3a). The results showed that the total number of white blood cells and lymphocytes in RAG2 KO pigs decreased significantly; eosinophils increased significantly; and basophils, neutrophils and monocytes increased significantly compared with the wild type. The results indicate that RAG 2 deficiency downregulates lymphocyte numbers. In order to further analyze the grouping structure of peripheral blood lymphocytes of RAG2 KO pigs, peripheral blood of three RAG2 KO pigs (P07, P11 and P12) was collected and PBMC cells were isolated, and WT pig PBMCs were used as the control group. CD3/CD45RA is a well-established marker for porcine mature B cells, which has already been reported by other studies [20,31]. In the WT group, the percentage of mature B lymphocytes (CD3^−^/CD45RA^+^) was 40.7%, while in the three RAG2 KO pigs, the percentages were 0.79%, 0.54% and 0.40% (Figure 3b,c). M/G and CD3 antibodies were used to label mature T cells. The results showed that the proportion of mature T cells (M/G^−^/CD3^+^) in WT PBMC cells was 34.0%, while in the three RAG2 KO pigs, the proportions were 0.92%, 0.81% and 1.60%, respectively (Figure 3d,e). These results showed that the proportion of mature B and T lymphocytes in RAG2 KO pigs was significantly reduced, which indicated immune cell dysplasia.

## 4. Discussion

Immunodeficient animals play an important role in research on the pathogenesis and treatment of human immunodeficiency diseases, as well as in the construction of tumor animal models and humanized animal models. RAG2 plays an important role in the V(D)J recombination process, and its deletion can impede lymphocyte differentiation in the early stage, resulting in the absence of mature T cells and B cells. The development of the pig immune system and the mechanisms of immune response are more similar to those of humans than mice, making pigs a useful tool for biomedical research. Generating the RAG2 KO immunodeficient pig animal model and studying its immune characteristics are of great significance for further exploring its application.

In this study, we generated RAG2 KO pigs by using CRISPR/Cas9 and SCNT technology. RAG2 KO fibroblast cells [29] were used as donor cells to obtain RAG2 KO pigs by somatic cloning technology. Two recipients gave birth to a total of twelve live piglets, with no stillborn piglets (Table 1). The successful generation of cloned individuals indicates that RAG2 KO does not affect the embryonic development of piglets. However, no further testing of embryonic development was carried out in this study, necessitating further experimentation. We used CRISPR/cas9 technology to generate the cloned individuals, whilst other research groups have also reported the use of TALENs technology to target the RAG gene of pigs and create immunodeficient pigs [32]. Among the 42 cloned piglets, 15 were stillborn, and the etiology of the elevated stillbirth rate remains unclear. However, off-target effects or aberrant reprogramming may be contributing factors to this anomaly. Regarding the survival of RAG1/2 gene-edited pigs, RAG1/2 biallelic knockout pigs grew slower than wild-type and heterozygous siblings and could not survive for more than 1 month under conventional feeding conditions [33]. The premature death of these homozygous pigs may have been due to immune-function defects. The longest survival time of RAG2 KO pigs in this study was 35 days, which was similar to previous studies. The long-term survival of immune-deficient pigs is still a problem that needs to be overcome.

The thymus, a crucial central immune organ in the human immune system, plays an indispensable role in immune function. As the primary site for T cell development, differentiation and maturation, the thymus significantly influences the efficacy of T cell responses [34]. In our study, the thymus of the RAG2 KO pigs was severely underdeveloped, and the thymus tissue was disorganized, with no obvious thymic corpuscles or germinal centers, indicating that RAG2 KO affected the development of thymic T lymphocytes (Figure 2a,b). The spleen is the largest secondary lymphoid organ in the human body and serves as a crucial hematopoietic and immune system component, rendering it one of the most significant organs in human physiology [35]. In this investigation, the spleen tissue structure of RAG2 KO pigs was disorganized, the central arterial duct in the red pulp of the spleen disappeared and the transition to the edge zone was blurred, indicating that RAG2 KO resulted in splenic dysplasia (Figure 2c,d). Studies have indicated that patients with SCID also exhibit symptoms of thymus and spleen dysplasia [36]. 

The hallmarks of SCID animal models are changes in immune cells, T and B lymphocytes, and NK cells. RAG1 and RAG2 KO mice also exhibit reduced T and B lymphocytes and impaired V(D)J rearrangement [7,8]. Rats with homozygous mutations in the RAG2 gene show a lack of mature T lymphocytes and B lymphocytes [37,38]. Studies on RAG gene function using pigs as experimental animals have shown that SCID pigs with RAG1/2 mutations also suffer from a lack of mature T and B lymphocytes and dysplasia of the spleen and thymus [39]. This study found similar results (Figure 2). B cells are pluripotent stem cells derived from human bone marrow. Their differentiation process can be categorized into five stages: pre-B cells, immature B cells, mature B cells, activated B cells and plasma cells. Immunoglobulin G (IgG) in serum is secreted by plasma cells [40]. RAG2 gene knockout resulted in a significant reduction in the number of mature B cells, and this would have impacted the production of plasma cells and consequently affected the secretion of IgG. Furthermore, our findings indicated a significant decrease in lymphocytes and an increase in eosinophil levels at one month of age in RAG2 pigs. Regrettably, due to the limited survival time of the RAG2 KO piglets, we were unable to obtain dynamic change results for blood white blood cells. The significant reduction in lymphocytes in peripheral blood is similar to previous reports [21,32] and was due to the inactivation of the RAG2 enzyme, which affects the development of T cells. It may be that the significant decrease in lymphocytes in peripheral blood, which reduced the immunity of the RAG2 KO piglets and caused some inflammatory reactions, caused a significant increase in eosinophils, replacing lymphocytes to compensate (Figure 3a). These may also have contributed to the reduced survival time of RAG2 KO pigs observed in this study.

## 5. Conclusions

In summary, we generated RAG2 KO pigs and evaluated the impact of RAG2 KO on the development of immune organs and immune cells. The thymus and spleen sizes of the RAG2 KO pigs were significantly smaller than those of WT pigs, and their structures were highly disorganized and devoid of notable characteristic structures, indicating that RAG2 KO leads to dysplasia of the organs. The proportion of mature T and B lymphocytes in the circulation of RAG2 KO pigs was significantly reduced as compared to WT pigs. These findings successfully verified the immunodeficiency phenotype of RAG2 KO pigs. This study may provide immunodeficient animals for the development of tumor models and humanized animals.

## Figures and Tables

**Table 1:** Generation of RAG2 KO pigs by somatic cell nuclear transfer in sows.

Recipients	No. of Transferred Embryos	Pregnancy (%)	Duration of Pregnancy (d)	No. of Offspring (Piglets)
1	660	+	116	6
2	660	-	-	-
3	680	+	116	6
4	680	-	-	-
Total	2680	2 (50%)		12

## Data Availability

The original contributions presented in the study are included in the article, further inquiries can be directed to the corresponding author.
